# Supplementary figures and images for: WISP2/CCN5 Suppresses Vasculogenic Mimicry through Inhibition of YAP/TAZ Signaling in Breast Cancer Cells
Source: Cancers (Basel). 2022 Mar 14;14(6):1487. doi: 10.3390/cancers14061487 (PMC8945957; doi:10.3390/cancers14061487)

Figure 1

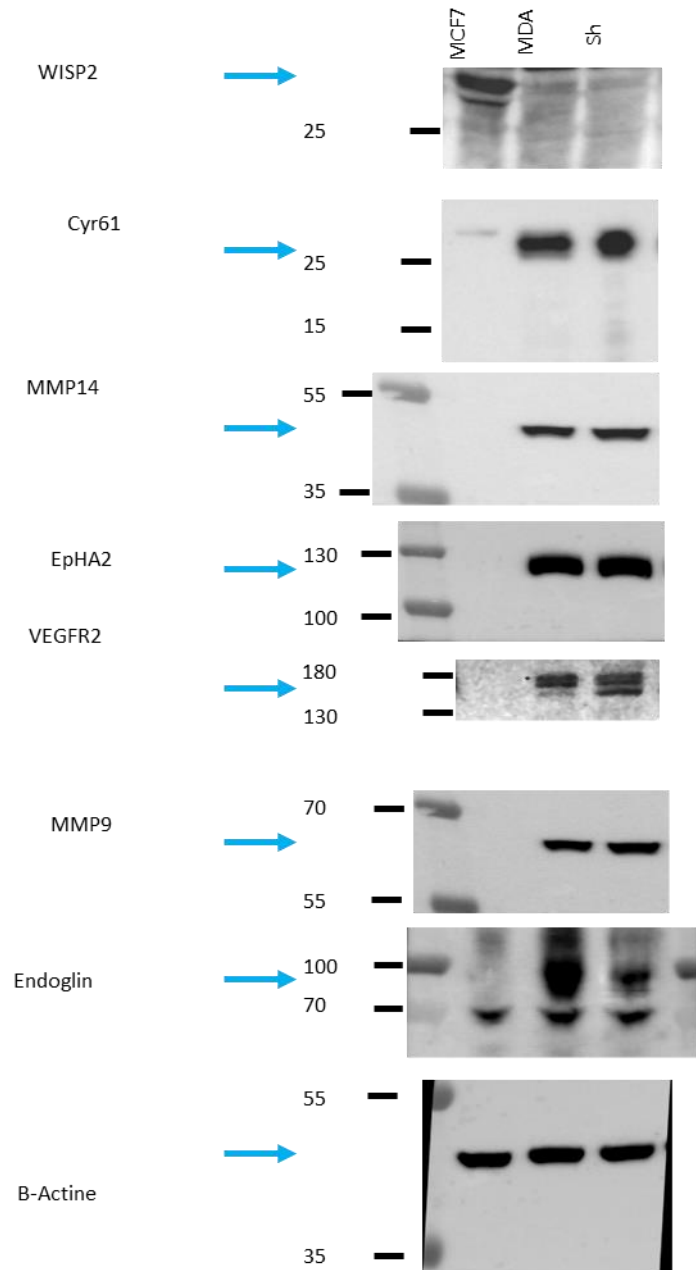

Figure 3

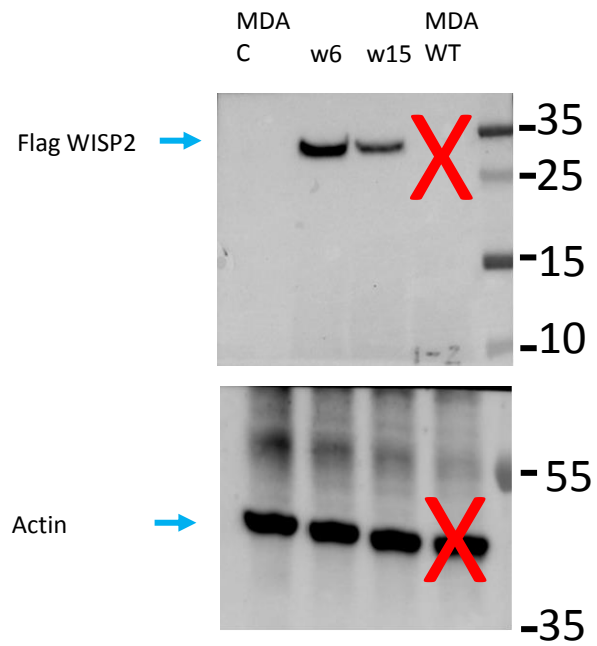

Figure 4B

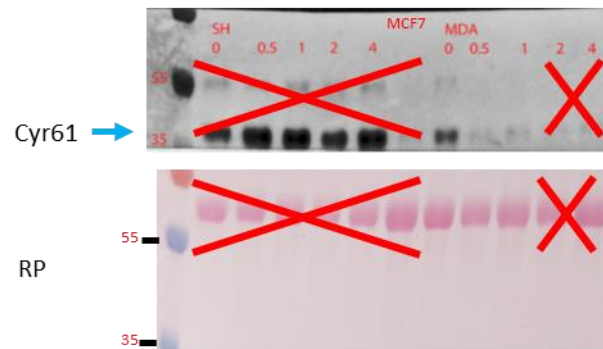

Figure 4C

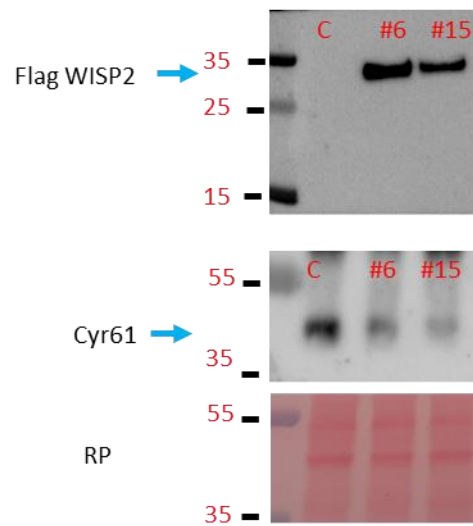

Figure 5A

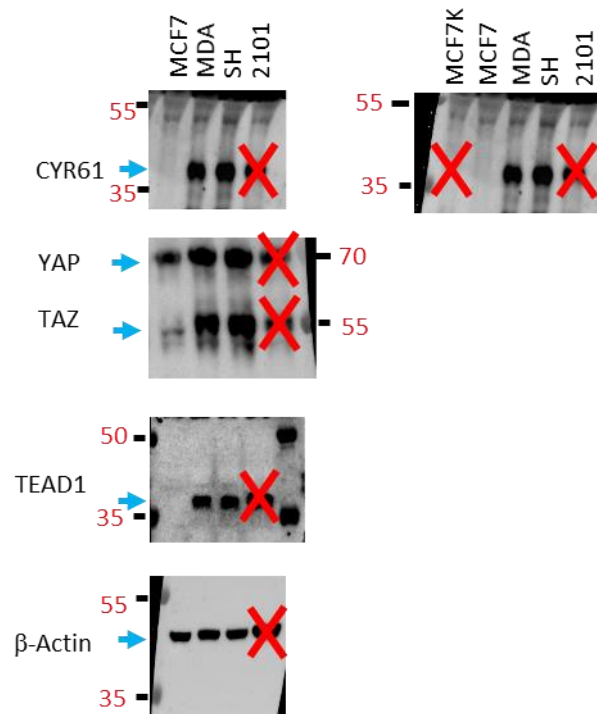

Figure 5C

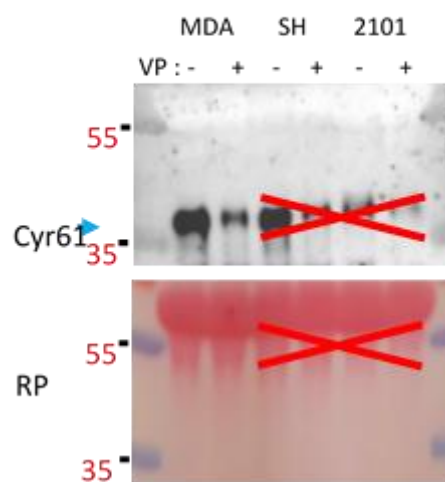

Supplement: Supplementary file 1 [file cancers-14-01487-s001.zip › cancers-1614394-file S1.pdf]

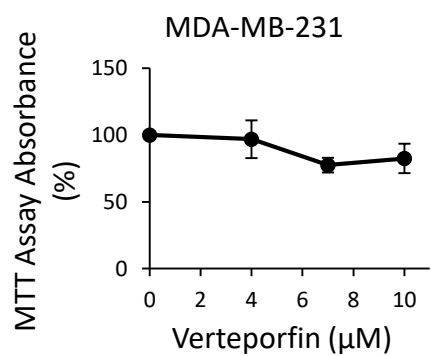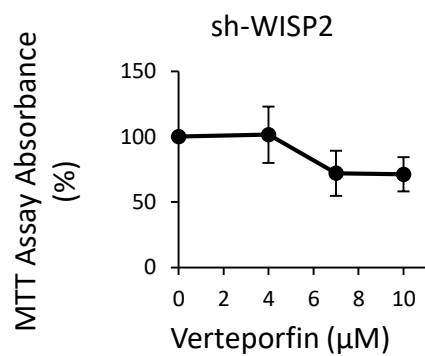

Supplement: Supplementary file 1 [file cancers-14-01487-s001.zip › Figure S1.pdf]
